# The global epidemiology of antibiotic-resistant *Neisseria gonorrhoeae*

**DOI:** 10.1093/jacamr/dlag245

**Published:** 2026-11-03

**Authors:** Matthew Ho, Paul C Adamson, Jeffrey D Klausner, Lao-Tzu Allan-Blitz

**Affiliations:** Columbia University Vagelos College of Physicians and Surgeons, New York, NY 10032, USA; Division of Infectious Diseases, Department of Medicine, School of Medicine, University of California Los Angeles, Los Angeles, CA, USA; Department of Population and Public Health Sciences, Keck School of Medicine, University of Southern California, Los Angeles, CA, USA; Division of Infectious Diseases, Department of Medicine, School of Medicine, University of California Los Angeles, Los Angeles, CA, USA

## Abstract

Antimicrobial-resistant *Neisseria gonorrhoeae* is an evolving global public health threat that increasingly limits treatment options for one of the world’s most common sexually transmitted infections. This review synthesizes the global epidemiology of antimicrobial resistance in *N. gonorrhoeae* across phenotypic, genomic, clinical and population-level dimensions. Resistance to agents no longer recommended for empiric treatment is widespread, while azithromycin resistance and reduced susceptibility to extended-spectrum cephalosporins continue to emerge. Resistant lineages, including internationally disseminated ceftriaxone-resistant and extensively drug-resistant strains, highlight the capacity of *N. gonorrhoeae* to acquire and spread resistance across geographic and genetic backgrounds. However, the global burden remains difficult to quantify because surveillance is uneven, extragenital and asymptomatic infections are underrepresented and susceptibility testing methods and interpretive thresholds vary across settings. Resistance is shaped by antimicrobial exposure, sexual network dynamics, international travel, delayed diagnosis and treatment and structural barriers to sexual healthcare. Opportunities to mitigate resistance include more representative and standardized phenotypic and genomic surveillance; diversified treatment strategies including aetiologic point-of-care diagnostics, rapid resistance-guided therapy and newly available oral agents and strengthened prevention efforts through partner notification and treatment and vaccine development. Coordinated, locally informed implementation of those approaches may help preserve effective treatment and reduce the impact of antimicrobial resistance.

## Introduction

There were an estimated 82 million new *Neisseria gonorrhoeae* infections worldwide in 2020.^1^ Those infections were disproportionately concentrated in low- and middle-income countries,^2^ though incidence has also risen in high-income settings in recent years.^3,4^ Of particular concern is the rapid emergence and spread of antimicrobial resistance, which threatens the effectiveness of first-line therapies. As a result, *N. gonorrhoeae* is an urgent global public health threat.^5,6^

The current understanding of global resistance patterns is largely derived from a patchwork of surveillance systems that collect phenotypic, genomic and other information about collected isolates. High-quality, representative surveillance data can not only detect emerging resistance early but also make treatment guidelines responsive to local epidemiology and monitor whether control measures are working.

In this review, we synthesize the global epidemiology of antimicrobial-resistant *N. gonorrhoeae* using phenotypic and genomic data. We examine the distribution of resistance determinants, the evolutionary and epidemiologic drivers shaping their transmission and the clinical and public health consequences of emerging resistance. We further highlight methodological challenges in current surveillance approaches and outline forward-looking strategies to strengthen monitoring, including integrating genomic epidemiology, expanding sentinel sites and developing novel diagnostics to predict resistance. Finally, we consider the evidence for etiologic point-of-care diagnostics, rapid resistance-guided therapy, novel antimicrobials and prevention strategies that all may help mitigate antimicrobial resistance and identify opportunities for targeted, scalable interventions.

## Existing surveillance systems

The largest and most comprehensive gonorrhoea surveillance programme is the World Health Organization (WHO) Gonococcal Antimicrobial Surveillance Program (GASP). It is a coordinated international laboratory network in which regional focal points aim to compile gonococcal antimicrobial susceptibility data from >100 representative isolates annually for each of the 77 participating countries/territories, organized into 6 regions: African, American, Eastern Mediterranean, European, SouthEast Asian and Western Pacific.^7,8^ An enhanced counterpart called the Enhanced Gonococcal Antimicrobial Surveillance Program (EGASP) was developed to strengthen sentinel surveillance by linking culture-based susceptibility testing to standardized clinical and epidemiologic data. It collects data from 46 sentinel sites and 19 laboratories in 12 countries (as of 2024), where men presenting with urethral discharge are consecutively identified.^9^ In 2024, 3615 isolates were collected from men, 64% of whom reported having sex with women only, 20% with men only and 5% with both.^9^

Regional systems have also arisen to fill gaps and provide more detailed information from selected areas. In Europe, the Euro-GASP collects and performs susceptibility testing on isolates from 24 European Union/European Economic Area Member States. In 2024, a total of 3371 isolates were analysed, 77% of which were genital samples with 46% of participants identifying as men who have sex with men.^10^ The USA also has its own Gonococcal Isolate Surveillance Project, which collects isolates monthly from the first 25 men with symptomatic urethral gonorrhoea at participating clinics. The enhanced counterpart extended that approach to the first 25 men or women with gonococcal infections of the pharynx or rectum and to up to the first 25 women with cervical or vaginal gonococcal infections who sought care at participating clinics.^11^ In the first 7 months of 2024, 1802 isolates were collected from male patients, 37% of which were men who have sex with men.^12^ Since late 2024, that project was incorporated into the Combatting Antimicrobial Resistant Gonorrhea and Other sexually transmitted infections (STIs) project, which has not released updated public reports at the time of this publication.

Those systems are essential because culture-based phenotypic assessment is the gold standard for antimicrobial susceptibility testing. However, nucleic acid amplification tests have become increasingly used for diagnosis, especially in high-income countries, due to their automation, speed, high sensitivity (>90%) and specificity (>98%),^13^ and lower cost. Historically, nucleic acid amplification tests have provided no information on antimicrobial susceptibility or on viable bacteria for culture, constraining antimicrobial resistance surveillance capacity.

Despite their importance, existing surveillance systems face several key limitations and biases. Geographic coverage remains uneven, with substantial gaps in Central America, the Caribbean, Eastern Europe, Africa, the Eastern Mediterranean and Southeast Asia.^8^ Some of those regions overlap with where the prevalence of infection is highest, in sub-Saharan Africa, Latin America and the Caribbean.^14^ Moreover, the reporting of significant isolates is highly geographically asymmetrical. Nearly half of ceftriaxone-resistant isolates with the mosaic *pen*A 60.001 allele were detected after 2022 in England and Australia,^15^ and the few dozen confirmed ceftriaxone treatment failures (defined as persistent infection after appropriate treatment, excluding reinfection) are reported almost exclusively in high-income countries with robust surveillance and diagnostics.^16^ That geographic distribution suggests significant underreporting of the true global prevalence of antimicrobial resistance.

Additionally, sampling frameworks may introduce bias and skew prevalence estimates. Many frameworks overrepresent symptomatic male urethral infections, while extragenital sites such as pharyngeal and rectal areas, along with infections in women, are under-sampled^17^ despite their potential as reservoirs for resistance or epidemiologic importance. Detection of asymptomatic infections is also often limited, especially in regions with restricted screening capacity, potentially underestimating the prevalence of infection. Moreover, sentinel sites are often STI clinics or specialty centres, where patients may seek treatment after visiting other healthcare providers. For instance, the 2024 WHO EGASP reports that in Indonesia, Thailand and Uganda, 28%, 16% and 15% of patients, respectively, reported antibiotic use within the past two weeks,^9^ which may cause an overrepresentation of resistant strains.

Finally, heterogeneity in laboratory methods,^18^ antimicrobial panels and interpretive breakpoints^19^ complicates cross-study comparisons and trend analyses. For example, standards development organizations report and define resistance differently (Table 1). The European Committee on Antimicrobial Susceptibility Testing (EUCAST) defines ceftriaxone resistance as a minimum inhibitory concentration (MIC) >0.125 mg/L and susceptible as MIC ≤0.125 mg/L.^20^ The WHO uses a decreased susceptibility threshold of MIC ≥0.125 mg/L^21^ and may report aggregated data with mixed thresholds.^8^ The Clinical and Laboratory Standards Institute (CLSI) recently adopted definitions of resistance as MIC ≥0.5 mg/L, intermediate susceptibility as MIC = 0.25 mg/L and susceptible as MIC ≤0.125 mg/L.^22^ Unless otherwise stated, we, in this review, define resistance as penicillin MIC >1 mg/L, tetracycline MIC >1 mg/L, ciprofloxacin MIC >0.064 mg/L, azithromycin MIC >1 mg/L and cefixime MIC ≥0.25 mg/L. We also define intermediate susceptibility for ceftriaxone as an MIC >0.125 mg/L. That cut-off was selected as the primary threshold for comparison because it permitted the greatest harmonization across datasets with differing susceptibility definitions and represents the more clinically relevant threshold at which clinicians would ideally defer use of ceftriaxone.

**Table 1.** Divergent ceftriaxone MIC Breakpoints for *N. gonorrhoeae* between EUCAST, WHO and CLSI

	Susceptible	Intermediate	Resistant	Decreased susceptibility
EUCAST	≤0.125 mg/L		>0.125 mg/L	
35th edition CLSI	≤0.25 mg/L			>0.25 mg/L
36th edition CLSI	≤0.125 mg/L	=0.25 mg/L	≥0.5 mg/L	
WHO	EUCAST		EUCAST or mixed^a^	≥0.125 mg/L

^a^The WHO GASP aggregated MIC thresholds of >0.125 mg/L (EUCAST breakpoint for resistance or updated CLSI breakpoint for intermediate susceptibility) or >0.25 mg/L (previous CLSI breakpoint for decreased susceptibility and updated CLSI breakpoint for resistance).

## Global distribution of phenotypes

Globally, resistance patterns vary by antimicrobial class but follow several consistent themes. Resistance to previously recommended agents is estimated to be widespread globally [trimethoprim/sulfamethoxazole (aggregation of EUCAST, CLSI and WHO thresholds), 42%; tetracycline (aggregation of EUCAST, CLSI and WHO thresholds), 45%^23^ and ciprofloxacin, 95%^9^], rendering these agents generally unsuitable for empiric therapy. Azithromycin resistance has an estimated global prevalence of 4.1%^9^ and is increasing, with 88% of countries reporting isolates with azithromycin resistance.^8^ Finally, resistance to cefixime and intermediate susceptibility to ceftriaxone are also increasing, with estimated prevalences of 11% and 5.2%,^9^ and 50% and 39% of countries reporting at least one isolate with resistance or intermediate susceptibility to cefixime and ceftriaxone, respectively.^8^ Notably, the 2024 EGASP report only included estimates for 12 sentinel countries: Brazil, Cambodia, Côte d’Ivoire, Indonesia, Malawi, Philippines, Qatar, South Africa, Thailand, Uganda, Vietnam and Zimbabwe.^9^ The estimates from the 2019–22 GASP report are more representative (from 77 countries), but aggregate different MIC thresholds (for ceftriaxone and cefixime: MIC >0.125 mg/L or MIC >0.25 mg/L).^8^

Multidrug-resistant strains are defined as resistant to ceftriaxone or azithromycin, plus at least two other antimicrobials (penicillin, tetracycline or ciprofloxacin). Extensively drug-resistant strains are defined by resistance to both ceftriaxone and azithromycin, plus at least two other antimicrobials (penicillin, tetracycline or ciprofloxacin).^24^ The existence of those phenotypes underscores the fragility of current therapeutic strategies and raises concern for the potential emergence of untreatable gonorrhoea.^25^ A recent review identified 33 probable cases of ceftriaxone treatment failure.^16^

Regional heterogeneity further shapes the global resistance landscape [Figure 1; Table S1 (available as Supplementary data at *JAC-AMR* Online)]. The areas with the highest prevalence of gonorrhoea include the WHO Western Pacific Region, Southeast Asian Region and African Region.^26^ Research has primarily centred on the WHO Western Pacific Region, where resistance to azithromycin, ceftriaxone and cefixime is increasing most rapidly. In 2019–22, the proportion of reporting countries in the Western Pacific Region with ≥5% intermediate susceptibility or resistance—the WHO-established action threshold for changing empiric treatment recommendations—was 40% for ceftriaxone, 50% for cefixime, 78% for azithromycin and 100% for ciprofloxacin.^8^ In the 2024 EGASP report, 99% of cefixime-resistant and cefixime-intermediate susceptibility isolates were found in Cambodia and Vietnam, with estimated prevalences of 8% and 24% for ceftriaxone and 60% and 38% for cefixime, respectively.^9^ Other reports have estimated even higher prevalences of ceftriaxone-intermediate susceptibility: up to 32% in Cambodia in 2021–22 and 28% in Vietnam in 2024.^27,28^ Similarly, azithromycin resistance rates were elevated in Cambodia (12%) and Vietnam (9%).^9^ In contrast, the Philippines and Indonesia had no significantly detectable resistance or intermediate susceptibility to ceftriaxone, cefixime, or azithromycin.^9^ Ciprofloxacin resistance rates were high across all countries examined: 99% in Cambodia, 98% in the Philippines and 99% in Indonesia.^9^

**Figure 1.**
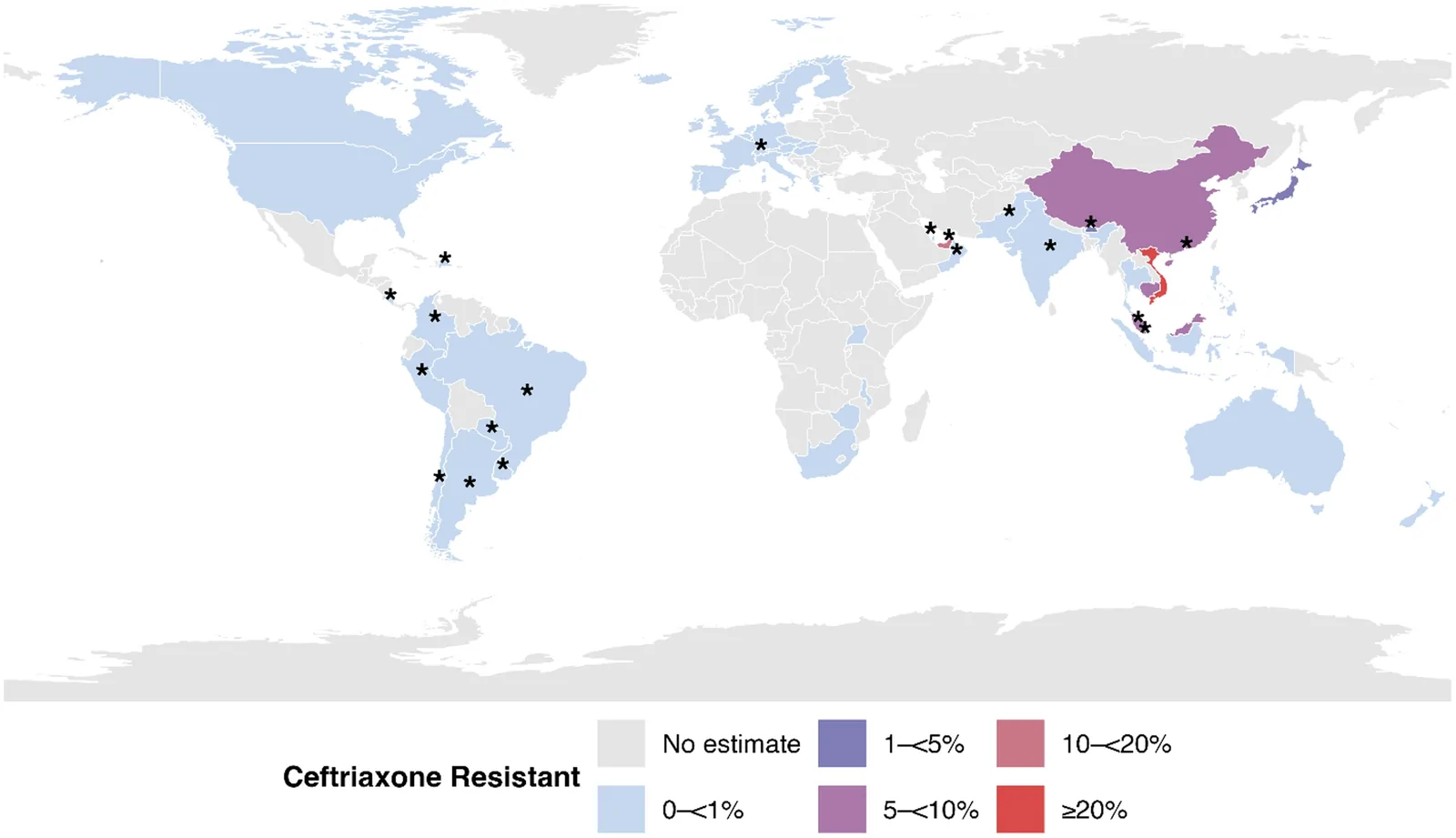
Global distribution of reported ceftriaxone resistance in *N. gonorrhoeae* from 2020 to 2024. The map shows the most recent eligible estimate (total number of isolates tested ≥40) for 56 countries. Data were sourced from the 2024 WHO EGASP, 2024 European Centre for Disease Prevention and Control (ECDC) Euro-GASP, 2019–22 WHO GASP (only data from 2020 onward were used) and other national surveillance reports (Table S1). For most of those studies, ceftriaxone resistance is defined as MIC >0.125 mg/L—both the EUCAST threshold for resistance and the updated CLSI threshold for intermediate susceptibility. Notable deviations include data from the 2019–22 WHO GASP, which are denoted with asterisks (Argentina, Bahrain, Bhutan, Brazil, Chile, Colombia, Costa Rica, Dominican Republic, Hong Kong, India, Malaysia, Oman, Pakistan, Paraguay, Peru, Singapore, Switzerland, United Arab Emirates and Uruguay). The WHO GASP aggregated MIC thresholds of >0.125 mg/L (EUCAST breakpoint for resistance or updated CLSI breakpoint for intermediate susceptibility) or >0.25 mg/L (previous CLSI breakpoint for decreased susceptibility and updated CLSI breakpoint for resistance); therefore, for countries included in that report using the prior CLSI breakpoint, strains with MIC = 0.25 mg/L would have been excluded, underestimating the true prevalence. All estimates should be interpreted and compared with caution because surveillance methods, study populations and antimicrobial susceptibility testing methods may differ across settings.

The Southeast Asia Region also has a high prevalence and diversity of resistance phenotypes. In 2019–22 GASP, the WHO estimated that among 3 reporting countries in the Southeast Asia Region, the proportion of reporting countries with ≥5% resistance is 33% for azithromycin and 100% for ciprofloxacin.^8^ In the 2024 EGASP, Thailand reported estimated prevalences of 94% for ciprofloxacin resistance, 1% for azithromycin resistance and 1% for cefixime resistance.^9^ Data from India from 2018 to 2022 estimated a prevalence of 13% for azithromycin-decreased susceptibility (MIC ≥1 mg/L), 2.2% for cefixime and 3.9% for ceftriaxone, along with 17 multidrug-resistant or extensively drug-resistant isolates.^29^ Estimates of prevalence in China in 2022 were 78% penicillin-resistant, 77% tetracycline-resistant, 98% ciprofloxacin-resistant, 17% azithromycin-decreased susceptibility (MIC ≥1 mg/L), 16% cefixime-resistant and 8.1% ceftriaxone-intermediate susceptible, with some regions reporting more than 25% ceftriaxone-intermediate susceptibility.^30^ Estimates from Japan in 2021 were 40% penicillin-resistant, 51% tetracycline-resistant, 75% ciprofloxacin-resistant, 11% azithromycin-resistant, 29% cefixime-resistant and 3.3% ceftriaxone-intermediate susceptible.^31^ Together, the Western Pacific and Southeast Asia regions have historically served as epicentres for the emergence and dissemination of resistant strains.^32^

In contrast, surveillance data from Africa and parts of the Eastern Mediterranean remain limited,^8^ though available evidence suggests substantial, likely underrecognized burdens of resistance.^14^ In 2019–22, the WHO estimated that the proportion of reporting countries in the Eastern Mediterranean Region with ≥5% intermediate susceptibility or resistance is 100% for ciprofloxacin, 40% for azithromycin, 67% for cefixime and 38% for ceftriaxone.^8^ Data from Qatar (part of the WHO Eastern Mediterranean Region) from 2017 to 2020 have estimated the prevalence of penicillin resistance (31%), tetracycline resistance (51%), ciprofloxacin resistance (65%), azithromycin resistance (4.1%) and ceftriaxone-intermediate susceptibility (1.0%).^33^ In the Africa Region, the 2024 WHO EGASP report estimated a high prevalence of ciprofloxacin resistance: 93% in Malawi, 100% in Uganda and 63% in Zimbabwe. Azithromycin resistance rates were 0% in Malawi, 4% in South Africa, 1% in Uganda and 2% in Zimbabwe. Resistance or intermediate susceptibility to cefixime and ceftriaxone was not detected.^9^

For the American and European Regions, surveillance data most often came from the US and European antimicrobial surveillance programmes. In the first 7 months of 2024, the USA reported antimicrobial resistance or intermediate susceptibility prevalences of 27% for penicillin, 38% for tetracycline, 46% for ciprofloxacin, 5.3% for azithromycin, 0.5% for cefixime and 0.1% for ceftriaxone.^12^ A review of data from Latin America for 1990–2018 estimated antimicrobial resistance (aggregation of CLSI and EUCAST thresholds) prevalences of 18%–98% for penicillin, 21%–90% for tetracycline, 6.0%–89% for ciprofloxacin, 32%–61% for azithromycin and 0.1%–8.5% for cephalosporins.^34^ The 2024 European programme reported antimicrobial resistance or intermediate susceptibility prevalences of 64% for ciprofloxacin, 19% for azithromycin, 0.3% for cefixime and 0.1% for ceftriaxone.^10^

Within regions, resistance is often concentrated in specific populations and sexual networks, including men who have sex with men, sex workers and individuals with high partner turnover.^35^ However, such nuances are not often collected from surveillance programmes. Those networks may also facilitate rapid dissemination of resistant clones and contribute to the persistence of high-level resistance phenotypes despite changes in treatment guidelines.^36^

## Genomic epidemiology of resistant *N. gonorrhoeae*

The previously discussed phenotypes are driven by a combination of chromosomally encoded mutations that alter antimicrobial targets, decrease drug influx or increase efflux pumps and plasmid-mediated mechanisms that enable drug inactivation or ribosomal protection. Sulphonamide resistance is conferred by mutations in *fol*P that reduce target binding^37^ and active efflux via *Mtr*F.^38^ Penicillin resistance has emerged through both plasmid-mediated production of β-lactamase (most commonly *bla*_TEM-1_) and chromosomal alterations in penicillin-binding proteins (*pen*A), often accompanied by reduced porin permeability (*por*B/*pen*B) and increased efflux (*mtr*R).^39^ Tetracycline resistance arises via ribosomal protection mediated by the often plasmid-encoded *tet*M determinant^40^ and chromosomal mutations in *rps*J that reduce target binding, *mtr*R mutations that yield increased efflux and *pen*B/*por*B mutations that reduce membrane permeability.^41^ Ciprofloxacin resistance is primarily mediated by a single mutation in codon 91 of the gyrase A (*gyr*A) gene that encodes DNA topoisomerase, which alters the ciprofloxacin binding domain.^42^ While other mutations in the *gyr*A and *par*C genes have been associated with ciprofloxacin resistance, *gyr*A S91F was found to be 98% sensitive and specific for ciprofloxacin resistance.^42^ Macrolide (azithromycin) resistance involves mutations in ribosomal genes like the 23S rRNA gene and upregulation of efflux systems, including *mtr*R promoter mutations.^43^ Reduced susceptibility to extended-spectrum cephalosporins is most strongly associated with mosaic *pen*A alleles—a patchwork of DNA fragments coming from different bacterial species, including commensal *Neisseria*, that thus has many scattered mutations that encode altered penicillin-binding proteins with diminished affinity for Β-lactams—often in combination with additional permeability and efflux adaptations.^44^

Interspecies recombination between *N. gonorrhoeae* and commensal *Neisseria* species in the oropharynx is believed to have led to the emergence of the mosaic *pen*A allele and may be a key factor in the emergence and spread of other novel resistance alleles.^45,46^ This recombination occurs predominantly in the pharynx, as (i) pharyngeal infections are typically asymptomatic and persist for a median of 16 weeks if untreated^47^ and (ii) provide increased opportunities for horizontal gene transfer of resistance alleles from commensal *Neisseria* species that naturally colonize the oropharynx.^46^ Moreover, ceftriaxone—the widely used drug of choice for *N. gonorrhoeae* infection—has poor penetration into pharyngeal tissues and saliva and is predominantly protein-bound,^48^ which can result in ineffective bacterial clearance. Beyond increasing the likelihood of treatment failure, sub-therapeutic antibiotic concentrations in the oropharynx can select for resistant strains in both *N. gonorrhoeae* and commensal *Neisseria* species.^49^ Similarly, that can occur with azithromycin, where, despite its excellent pharyngeal tissue concentration, its prolonged half-life leads to extended durations of sub-inhibitory concentrations in the oropharynx for up to 4 weeks after treatment.^48^

To detect genomic alterations that may underlie antibiotic resistance phenotypes, multiple targeted sequence-based typing schemes have been used to classify *N. gonorrhoeae* strains. Historically, Multilocus Sequence Typing (MLST) and *N. gonorrhoeae* Multi-Antigen Sequence Typing (NG-MAST) have classified strains using housekeeping genes (*abc*Z, *adk*, *aro*E, *fum*C, *gdh*, *pdh*C, *pgm*)^50^ and highly variable genes (*por*B and *tbp*B),^51^ respectively. Those schemas, however, do not provide sufficient resolution or accuracy for molecular epidemiology of antibiotic resistance.^52^  *N. gonorrhoeae* Sequence Typing for Antimicrobial Resistance (NG-STAR), which uses gene sequences associated with antibiotic resistance (*pen*A, *mtr*R, *por*B, *pon*A, *gyr*A, *par*C and 23S rRNA),^53^ is better suited for antimicrobial resistance surveillance. More recently, whole-genome sequencing-based approaches have become increasingly possible and provide much finer resolution for defining transmission clusters and linking lineages to specific resistance determinants. For example, core genome MLST uses 1668 loci critical to the gonococcal genome.^54^ Even newer systems include Life Identification Number codes that use 1430 genes from the core genome MLST.^55^

Those approaches have consistently shown that resistance is concentrated in a limited number of globally disseminated genetic backgrounds, even as local outbreaks and country-specific clades continue to emerge. The genomic epidemiology of resistant *N. gonorrhoeae* reflects successful, recombining lineages rather than a single globally dominant clone.

Among the best characterized resistant lineages is MLST ST1901 (NG-MAST G1407/NG-STAR CC90/core genome MLST cgc200). The ancestor of those clones emerged in East Asia around 1944, predating the introduction of cephalosporins.^56^ It subsequently dispersed from Asia in two waves in 1987 and 1996. The ancestor of the second wave acquired the mosaic *pen*A-34.001 allele, which reduces susceptibility to cephalosporins and has become established as a globally dominant multidrug-resistant lineage.^32^ Beyond reduced cephalosporin susceptibility, the strain is also resistant to ciprofloxacin and increasingly resistant to azithromycin.^57^

Another major lineage is FC428 (MLST ST1903/NG-MAST ST3435/NG-STAR ST233), first identified in Japan in 2015 and notable for its mosaic *pen*A allele 60.001, which is linked to ceftriaxone resistance.^58^ Beyond cephalosporin resistance, the strain is also resistant to penicillin, tetracycline and ciprofloxacin. FC428 and FC428-like strains have since been reported across Asia,^58,59,60,61^ Europe,^62,63^ Oceania^64^ and North America,^65^ with phylogenetic studies suggesting multiple introductions and ongoing diversification after dissemination. Newer descendants of that strain are beginning to acquire azithromycin resistance.^61,66^

A third widely discussed clone is A2543 (MLST ST16406), the first identified extensively drug-resistant lineage with resistance to both cephalosporins from the penA-60.001 allele and azithromycin from the A2059G mutation in 23S rRNA, as well as to ciprofloxacin, tetracycline and penicillin. It was first identified in 2018^67^ and has since expanded internationally,^68,69,70,71^ with Southeast Asia likely serving as the primary geographic reservoir.^72^

Overall, the greatest genomic diversity and the origin of highly resistant clones have been repeatedly reported in the Western Pacific and Southeast Asia, regions that appear to serve as long-standing reservoirs and hubs for disseminating resistant gonococci. Cephalosporin-resistant or cephalosporin-reduced-susceptibility strains are often linked to mosaic *pen*A backgrounds, like *pen*A-34, *pen*A-60 and recently *pen*A-237.^73,74^

## Drivers of resistance and the epidemiology of those drivers

Antimicrobial resistance in *N. gonorrhoeae* is shaped by a layered set of clinical, behavioural and population-level forces that accelerate selection and dissemination (Table 2). At the programmatic level, syndromic management remains an important driver in many low- and middle-income countries, where individuals with symptoms of a sexually transmitted disease are treated empirically for likely pathogens causing that syndrome.^75,76^ While that approach is necessary in settings with limited diagnostic capacity, it can promote antimicrobial resistance by increasing unnecessary antibiotic exposure among symptomatic patients due to low diagnostic specificity (5.6%–33% in a study in Uganda).^77^ By definition, syndromic management will also not address asymptomatic infections that may continue to transmit and maintain undetected reservoirs of infection, such as in extragenital sites relevant to the emergence of gonococcal resistance.

**Table 2.** Drivers of resistance in *N. gonorrhoeae*, the mechanisms by which they select for resistance and interventions to control, reduce and mitigate resistance

Drivers of resistance	Mechanism of selection	Interventions to control, reduce and mitigate resistance
Syndromic management (treatment of a clinical syndrome with a standard regimen covering likely pathogens, without aetiology confirmation)	Unnecessary antibiotic exposure and selection for resistance	Shift from syndromic to aetiologic testing where possible Support development of inexpensive point-of-care diagnostic tests
Repeated exposure to shrinking therapeutic options	Sustained selection pressure on a last-line agent	Diversify treatment regimens by using resistance-guided therapy or locally informed treatment recommendations Support development and positioning of novel agents (zoliflodacin and gepotidacin), coupled with stewardship and resistance surveillance
Bystander selection (selection for antibiotic resistance in a microorganism exposed to antibiotics prescribed for another indication)	Selection for antibiotic resistance	Antimicrobial stewardship Strengthen prescription regulations Reduce unnecessary over-the-counter access
Structural barriers, delayed diagnosis/limited diagnostic infrastructure, incomplete treatment, inadequate follow-up	Persistent infection and onward transmission	Improve access to sexual health services Strengthen laboratory infrastructure and affordable diagnostics Ensure treatment completion, improve follow-up and provide test-of-cure where clinically indicated
Dense sexual networks, high partner turnover and concurrency	Rapid dissemination of successful resistant lineages	Offer timely partner notification and treatment Co-design voluntary, confidential, non-stigmatizing prevention testing and surveillance in communities experiencing higher transmission
International travel and migration	Dissemination of resistant clones	Investigate suspected travel-associated outbreaks and coordinate public health responses Rapid international sharing of resistance data
Asymptomatic and extragenital infection	Undetected reservoirs and prolonged transmission	Expand culture and nucleic acid amplification test surveillance to pharyngeal and rectal sites Improve detection of asymptomatic infection while avoiding indiscriminate screening that may increase antibiotic exposure
Pharyngeal infection	Prolonged persistence, limited ceftriaxone penetration and opportunity for horizontal gene transfer with commensal *Neisseria*	Improve pharyngeal diagnosis and test-of-cure strategies Support development of effective non-ceftriaxone options for pharyngeal infection Intensify surveillance of resistance markers via whole-genome sequencing for pharyngeal infections
Uneven surveillance and culture capacity	Delayed detection and biased prevalence estimates	Expand sentinel surveillance, rebuild culture capacity, standardize antimicrobial susceptibility testing, integrate whole-genome sequencing Improve representation of women, asymptomatic infections and extragenital sites
High gonorrhoea incidence/reinfection	Increased opportunities for selection and transmission	Combine diagnosis, effective treatment, partner services, prevention and surveillance Support development of longer-term preventive approaches including a gonococcal vaccine

Even in higher-resource settings, a one-size-fits-all treatment paradigm contributes to resistance, as demonstrated by the successive emergence of resistance in *N. gonorrhoeae* to first-line agents throughout its history. That challenge is amplified by the shrinking therapeutic arsenal for gonorrhoea, with most international guidelines now recommending increasing doses of ceftriaxone monotherapy for the treatment of every case of gonorrhoea. Delayed diagnosis, incomplete treatment, poor follow-up and variable access to high-quality sexual health services further increase the likelihood that partially suppressed infections persist long enough to transmit resistant strains.

Antibiotic exposure outside gonorrhoea treatment is also important for resistance evolution. Prior use of ciprofloxacin, macrolide and cephalosporin antibiotics for unrelated infections can select for resistant gonococcal populations at the community level, especially where these agents are widely prescribed or easily obtained.^78,79,80^ That bystander selection is particularly important for azithromycin and ciprofloxacin, which have historically been used broadly in outpatient care.^81,82,83^

Stewardship practices also vary substantially by region and may affect gonococcal resistance. Across antibiotic-resistant bacteria in general, antimicrobial stewardship programmes have demonstrated effectiveness across several species and antibiotics.^84,85,86^ However, the effectiveness of antimicrobial stewardship against *N. gonorrhoeae* specifically is more uncertain and may depend on the antibiotic and local epidemiologic context. In the USA and UK, removal of cefixime as a first-line therapy was associated with a decline in isolates with elevated cefixime MICs.^87,88^ In contrast, discontinuing penicillin or fluoroquinolones did not lower the prevalence of resistance to these drugs in the USA,^89^ potentially because resistance determinants carry little or no fitness cost and/or because of ongoing bystander selection from continued use of those agents for non-gonococcal indications.^90^

The impact of stewardship may also depend on the relative contributions of domestic antibiotic use and international importation to resistance emergence. As discussed in previous sections, highly resistant strains have spread from the Western Pacific and Southeast Asia to the rest of the world. That importation plays an important role in resistance emergence in the USA^91^ and other high-income countries. Where importation is a dominant driver, domestic stewardship alone may have limited impact on resistance trends. Conversely, in regions where local antibiotic use and sustained transmission are the primary drivers, stewardship might have greater effect. The extent to which antimicrobial stewardship can reduce gonococcal resistance may therefore vary substantially across settings. Definitive evidence remains limited, particularly in countries with a high burden of antimicrobial-resistant *N. gonorrhoeae*, where stewardship interventions and their potential impact on resistance warrant further investigation.

A relevant, recent example of how antibiotic exposure guides resistance evolution is the expansion of *tet*M after the adoption of post-exposure doxycycline prophylaxis. In the USA, *tet*M prevalence increased from below 10% in 2020 to >30% in the first quarter of 2024, coinciding with the shift away from azithromycin and towards doxycycline for the treatment of chlamydia.^92^ In Southern California, doxycycline post-exposure prophylaxis rapidly lost effectiveness against gonorrhoeae within its first year after implementation. That was driven predominantly by an expansion in *N. gonorrhoeae* lineages harbouring the *tet*M gene.^93^ Similar results were also reported in France and Italy.^94,95^

Other potential exposures that could contribute towards antibiotic resistance include low-level environmental exposures. Ongoing modelling work suggests that background environmental exposure can offset the fitness costs of resistance and prevent reversion to antibiotic-susceptible variants, even for human-host-restricted pathogens like *N. gonorrhoeae*.^96^

Sexual network dynamics strongly influence how resistant strains spread once they emerge. High partner exchange rates, concurrency and densely connected sexual networks increase the probability that a successful resistant lineage will disseminate rapidly.^36,97^ Travel-associated sexual exposure can further seed resistant clones across cities, countries and continents, allowing local outbreaks to become international transmission events.^98,99^ Core groups of individuals at increased risk for repeat infection, such as men who have sex with men, sex workers and their clients, are also more frequently exposed to antibiotics; in tandem with dense and interconnected sexual networks, those forces can amplify transmission of resistant gonococcal strains.^35^

Screening further influences when resistant strains are detected and treated. For urogenital gonorrhoea infections, around 80%–90% of women and 50%–90% of men are asymptomatic. For extragenital infections, 50%–100% of both men and women are asymptomatic.^100^ Hence, reliance on symptom-based care allows a substantial reservoir of infection to go undetected, untreated and thereby spread. A modelling study in a closed cohort of men who have sex with men showed that quarterly *Chlamydia trachomatis* and *N. gonorrhoeae* screening can detect asymptomatic infections earlier and has the potential to reduce transmission.^101^

However, evidence against screening also exists. Studies from Belgium and the Netherlands found no population-level benefit from asymptomatic screening among individuals at increased risk for repeat infections and increased resistance in *N. gonorrhoeae*.^102,103^ Moreover, the relationship between screening and resistance is complex. More frequent, comprehensive screening detects more infections, which in turn increases treatment and antibiotic exposure and strengthens selection pressures for resistance among individuals likely to experience reinfection. Notably, a Belgian network model found that intensive screening in men who have sex with men with fewer than 15 casual partners per year had little effect on gonorrhoea prevalence but increased the probability of azithromycin-resistant gonorrhoea emergence. In contrast, intensive screening in men who have sex with men with more than 15 casual partners per year reduced both prevalence and macrolide resistance.^104^ Further direct studies on that relationship are needed.

Structural and social determinants create the environment in which the epidemiologic forces operate. Urbanization increases sexual network connectivity and facilitates faster spread of resistant clones.^105^ On a global scale, travel and migration may also introduce resistant strains into new populations.^98,99,106^ Historically, commercial sex work was a major driver of the dissemination of penicillin- and other antibiotic-resistant *N. gonorrhoeae* strains, with self-prescribed prophylactic antibiotic use strengthening selective pressures and consequently high rates of resistance.^107,108^ More recently, men who have sex with men have become an important group in the spread of *tet*M-expressing, ciprofloxacin-resistant and extended-spectrum cephalosporin-resistant strains.^35^ Criminalization, stigma and discrimination against sex workers and same-sex sexual behaviours further discourage testing, disclosure and partner services, thereby allowing infections to persist and spread unnoticed in important populations.^109,110^ In many settings, those social determinants of health overlap with limited laboratory infrastructure and inconsistent access to effective antibiotics, creating a feedback loop in which resistance is both selected for and poorly contained.

## Consequences of antimicrobial resistance in *N. gonorrhoeae*

Increasing antimicrobial resistance in *N. gonorrhoeae* has undermined the effectiveness of standard treatment, causing rising treatment failures.^16,111,112,113,114,115^ When first-line regimens fail, patients often require repeat testing and retreatment with alternative agents, thereby delaying cure and increasing the risk of onward transmission. If adequate treatment is not received, as can occur in low- and middle-income settings, persistent *N. gonorrhoeae* infection can progress to complications including pelvic inflammatory disease,^116^ infertility,^117^ ectopic pregnancy,^118^ premature birth,^119^ epididymitis^120^ and an increased risk for the acquisition and transmission of human immunodeficiency virus.^121,122^ In neonates, maternal infection increases the risk for blindness,^123^ disseminated infection, other serious complications and even perinatal mortality.^119^ As resistance narrows treatment options, the risk of infections going untreated or only partially treated increases, with greater consequences for patients already facing barriers to timely care. Modelling estimated that gonococcal infection caused the loss of 386 lifetime quality-adjusted life years among women and 12 112 among men in the USA in 2018.^124^ Increasing treatment failures of gonorrhoea will only cause that figure to increase.

At the population level, antimicrobial resistance increases healthcare utilization and costs through persistent infections, management of complications and laboratory testing.^125^ In the USA, the costs associated with antimicrobial-resistant gonococcal infections were $214.5 million in 2023.^121,126^ Antimicrobial resistance also affects the cost-effectiveness of control strategies. In higher-income settings, healthcare systems can adopt intensified surveillance/screening, test-of-cure programmes,^127^ and increased reliance on injectable-only therapies (which require administration in a healthcare facility, compared with oral therapies that can be prescribed) to initially address higher levels of antibiotic resistance.^128,129,130^ However, over time, those interventions will strain limited public health resources. Such costs also reflect system-level pressures on sexual health services that must manage higher patient volumes with fewer reliably active drugs.

Hence, the asymmetrical prevalence of antibiotic-resistant gonorrhoea results in an unequal burden on patient populations. Generally, populations that have restricted access to effective diagnostics and treatments are more likely to experience delayed recognition of resistant gonorrhoea and poorer outcomes. Moreover, the areas with strained sexual healthcare systems also tend to be those where surveillance is most limited. Hence, antimicrobial resistance amplifies existing inequities in sexual and reproductive health.

## Advancing surveillance, diversifying treatment and strengthening prevention

Advancing surveillance, diversifying treatments and strengthening prevention will be central to slowing the global spread of antibiotic-resistant *N. gonorrhoeae*. A priority is to strengthen surveillance architecture by expanding and harmonizing regional and global systems so data from disparate settings can be compared more reliably and closer to real time. At present, surveillance remains uneven across countries and heavily weighted towards well-resourced health systems, limiting the precision of global estimates and obscuring emerging resistance in underrepresented regions.^15,48^ Better integration of whole-genome sequencing into routine surveillance would substantially improve outbreak detection, lineage tracking and identification of resistance determinants.^52,54^ Sequencing should be linked with standardized epidemiologic metadata and, where possible, with clinical outcome data.^48^ Equally important is improving capture of extragenital infections and infections among key populations,^8^ since pharyngeal and rectal sites are often under-sampled despite their role in transmission and the emergence of resistance.^131,132^ Shared analytic platforms and more timely data exchange across laboratories, public health agencies and clinical networks would help convert surveillance from a descriptive exercise into an effective control strategy.

Treatment diversification is also crucial to slowing the emergence and spread of antimicrobial resistance in *N. gonorrhoeae*. Rather than relying on a one-size-fits-all treatment approach, diversification involves tailoring therapy to the infection and the susceptibility of the infecting strain through three complementary strategies: (i) shifting from syndromic to etiologic treatment (aetiology-based treatment), (ii) using rapid resistance-guided diagnostics to direct therapy with existing antimicrobials (resistance-guided therapy) and (iii) introducing new therapeutic agents (novel antimicrobials). By reducing unnecessary exposure to individual antimicrobials and expanding the range of agents used to treat *N. gonorrhoeae* infection, those approaches may reduce selection pressure for resistance while preserving treatment options for future infections.^133^

To advance aetiology-based treatment, low-cost, point-of-care tests for *N. gonorrhoeae* are increasingly available and have demonstrated high sensitivity and specificity, especially for urogenital infections. Clinical trials and implementation studies have shown that those technologies reduce overtreatment and undertreatment, reduce loss to follow-up and shorten time to treatment.^134^ Application of point-of-care technologies to shift syndromic management towards etiologic treatment in resource-limited settings has been shown to improve timelines and accuracy.^135,136^ Further implementation research is necessary to inform deployment given supply and cost constraints.^137^

Additionally, rapid tests that can predict resistance can allow clinicians to tailor therapy at the time of diagnosis—also known as resistance-guided therapy—rather than relying on empiric regimens alone.^138^ Molecular assays detecting the *gyr*A S91 can identify infections susceptible to ciprofloxacin with high sensitivity and specificity. Across diverse settings, studies have demonstrated >97% cure rates with ciprofloxacin when guided by rapid molecular resistance tests as well as real-world reductions in ceftriaxone use.^139,140,141^ In Seattle, reflex *gyr*A had a mean turnaround time of 2.8 days, leading to 26% of gonorrhoea-positive patients successfully treated with ciprofloxacin with negative tests of cure.^141^ Modelling supports that use of rapid resistance tests will delay the emergence of ceftriaxone resistance,^142,143,144^ while a point-of-care *gyr*A test would introduce additional benefits of reduced losses to follow-up and reduce the time to treatment of asymptomatic infections.^145^

Continued discovery of other molecular markers and emerging resistance mechanisms relies on continued culture and phenotypic susceptibility testing. Hence, culture capacity should be rebuilt, and standardized susceptibility testing should remain a core component of surveillance.^18,146,147^ Moreover, developing locally oriented and owned surveillance systems would facilitate individualized treatment strategies using local resistance patterns and diagnostic data to guide therapy where possible.^148^

Novel antimicrobials are also becoming available. Zoliflodacin and gepotidacin have completed Phase 3 evaluation and have been approved by the US Food and Drug Administration for uncomplicated urogenital *N. gonorrhoeae* infection. They have been shown to be non-inferior to ceftriaxone plus azithromycin, with high microbiological cure rates.^149,150^ Implementation of newly available oral therapies offers a crucial opportunity to broaden the treatment pipeline and reduce reliance on ceftriaxone as the primary treatment for gonorrhoea.^151^ By providing effective alternatives to ceftriaxone, zoliflodacin and gepotidacin could reduce the proportion of infections treated with ceftriaxone, thereby decreasing selection pressures favouring ceftriaxone-resistant strains.

However, both agents are themselves vulnerable to resistance. Gepotidacin has documented in-treatment resistance selection^152^ and ∼12% circulating non-susceptibility in Hangzhou, China, a high-resistance region.^153^ Zoliflodacin resistance is so far confined to laboratory-generated mutants and transformation experiments.^153,154,155,156^ Cross-resistance between the two agents, however, has been identified through the *gyr*B D429N mutation and links the two drugs’ futures.^157^ Their long-term value will therefore depend on evidence-based deployment strategies, susceptibility-guided treatment and continuous phenotypic and genomic surveillance to detect emerging resistance early.^158^

Important gaps remain in defining the optimal placement of zoliflodacin and gepotidacin within treatment algorithms. Modelling studies evaluating strategies for introducing new antibiotics to treat *N. gonorrhoeae* suggest that early introduction through equal allocation or combination therapy can delay resistance compared with sequential or reserve strategies.^159,160^

Additionally, ensuring access across settings is a concern. Availability may initially be limited to countries where regulatory approval and distribution have been established, while the cost of new agents may create additional barriers to adoption in low- and middle-income countries and other resource-limited settings. Zoliflodacin may partially mitigate those barriers through the Global Antibiotic Research and Development Partnership’s not-for-profit access model, which incorporates rights for registration and supply across many low- and middle-income countries and development efforts to lower manufacturing costs.^161^ However, its eventual affordability and availability will still depend on country-specific pricing, regulatory approvals, procurement and distribution capacity.

Finally, data remain limited on whether those new agents can effectively treat extragenital disease, with some concern that zoliflodacin is less effective than ceftriaxone for treating pharyngeal gonorrhoea.^162^ Hence, development of alternative therapies specifically for pharyngeal gonorrhoea beyond ceftriaxone remains critical. As noted before, the pharyngeal site is important given rising resistance rates, the proposed importance of horizontal gene transfer in the pharynx and the lack of effective alternative agents.^45,163^ Recently, deep learning models have helped discover novel, effective antibiotics against *N. gonorrhoeae*.^164^

After infection detection and treatment, partner notification and therapy strategies should also be strengthened. Modelling and empirical studies support that increasing coverage and timeliness of partner notification systems can reduce infection incidence.^165,166,167^ In nearly all US states, expedited partner therapy—treating sex partners from the prior 60 days of a patient diagnosed with an STI by giving the patient medication or a prescription to deliver to the partner without the provider examining that partner—has been implemented.^91^ A systematic review of clinical trials showed that those programmes reduce reinfection and prevalence at follow-up.^168^ A few studies have reported promising results in other nations,^169,170^ though legal and regulatory barriers remain a concern, and more robust data are needed. Studies characterizing the effect of partner treatment programmes on the development of antibiotic resistance are also lacking. Additionally, experience from the Strengthening the US Response to Resistant Gonorrhoea programme refines how resources should be invested for maximum effect. Substantially more resource-intensive enhanced partner services (consisting of interviewing and testing of sexual partners of partners who tested negative for *N. gonorrhoeae* and social contacts of index patients and partners) identified only three additional diagnoses compared with traditional partner services.^171^ Those findings emphasize that detailed estimates of the clinical and economic consequences are needed to guide investment decisions. Specifically, strengthening established partner notification services may offer a more scalable approach than indiscriminately expanding resource-intensive investigations.

Ultimately, the creation of a gonococcal vaccine would be a sustainable solution.^172,173,174^ Early observational studies showed that the 4CMenB vaccine provided moderate cross-protection in the general population against *N. gonorrhoeae*, with pooled vaccine effectiveness estimates of approximately 32%–41% across studies and meta-analyses.^175,176,177^ However, more recent randomized controlled trials enrolling men who have sex with men showed no significant protective effect.^178,179^

Several research gaps remain especially important. More robust, standardized estimates of the clinical and economic consequences of resistance are needed to guide investment decisions and quantify the benefits of stronger surveillance and treatment programmes.^125^ Predictive models that forecast resistance trajectories across different treatment and stewardship scenarios would help health systems plan for future threats rather than react after resistance becomes widespread.^180^ Finally, the field needs continued investment in novel therapeutics and in studies that evaluate their real-world impact, including how quickly resistance emerges after their introduction and how new drugs should be positioned within broader control strategies. Those interventions need evaluation not only for microbiologic effects but also for acceptability, feasibility, equity and cost.

### Conclusion

Antimicrobial resistance in *N. gonorrhoeae* is an evolving and urgent threat to global public health. It has eroded many historical treatment options and continues to expand across geographic and genetic backgrounds. Resistance is driven by interrelated clinical, behavioural and population-level factors, while substantial surveillance gaps—particularly in low-resource settings and among extragenital and asymptomatic infections—limit understanding of its true burden. However, the trajectory is not solely one of therapeutic decline. Partner notification and treatment programmes have shown measurable effects on transmission; point-of-care diagnostics can shift treatment away from syndromic management; molecular resistance testing can diversify treatment beyond ceftriaxone and two new oral antimicrobials now expand therapeutic repertoire for uncomplicated urogenital infections. Durable control will nevertheless require more representative surveillance, standardized susceptibility testing, integrated genomic surveillance, expanded and equitable implementation of diagnostics and resistance-guided therapy, sustained culture capacity, responsible introduction of new therapeutics and continued investment in prevention, including vaccine development. Progress may be achieved by coordinating interventions with demonstrated or emerging effectiveness, followed by evaluation of their real-world impact across different epidemiologic and health-system contexts.

## Supplementary Material

dlag245_Supplementary_Data

## Supplementary data

Table S1 is available as Supplementary data at *JAC-AMR* Online.
